# Does critical care paramedic deployment improve delivery of post-resuscitation care following out-of-hospital cardiac arrest? A retrospective cohort study

**DOI:** 10.29045/14784726.2025.6.10.1.1

**Published:** 2025-06-01

**Authors:** Alan Cowley, Dan Cody, Paul Rees

**Affiliations:** South East Coast Ambulance Service NHS Foundation Trust ORCID iD: https://orcid.org/0000-0002-3093-4395; South East Coast Ambulance Service NHS Foundation Trust; Barts Heart Centre; The Blizard Institute ORCID iD: https://orcid.org/0000-0002-6560-6332

**Keywords:** cardiac arrest, pre-hospital, ROSC, specialist paramedic

## Abstract

**Introduction:**

The return of spontaneous circulation (ROSC) care bundle is a set of interventions designed by NHS England for use in the post-resuscitation care of patients following out-of-hospital cardiac arrest (OHCA). Compliance with these standards is critical in providing optimal, standardised care and in improving outcomes. This study aimed to investigate the impact of critical care paramedics (CCPs) on delivery of post-ROSC care.

**Methods:**

A retrospective observational study was conducted across a large UK ambulance service. All patients with sustained ROSC following resuscitation for OHCA over a one-year period were included. The post-ROSC care delivered to two groups was compared – a standard care group, and a group where a CCP was present.

**Results:**

The study included 997 incidents: 106 incidents in the non-CCP group and 891 incidents in the CCP group. The presence of a CCP was associated with a statistically significant increase in compliance with the ROSC bundle. Of incidents with a CCP present, 75% were fully compliant, compared with 64% of incidents without a CCP. The mean percentage compliance across the standards was significantly higher in the CCP group. Secondary outcome analysis showed statistically significant benefits in compliance for several care parameters when a CCP was present.

**Conclusion:**

This retrospective study confirms that the presence of a CCP improves delivery of post-ROSC care. This highlights the potential benefits of having CCPs as part of the standard pre-hospital care resuscitation team. Further research is needed to confirm these findings and to examine the relationship between the ROSC bundle and patient outcomes.

## Introduction

Neurologically intact survival is the ultimate desired endpoint when treating someone in cardiac arrest. However, this cannot be achieved without an initial return of spontaneous circulation (ROSC). In the UK, for the year ending September 2022, 25% of out-of-hospital resuscitation attempts resulted in ROSC on arrival at the hospital. This percentage rises to 46% in the ‘Utstein’ comparator group, which includes patients with an out-of-hospital cardiac arrest (OHCA) of presumed cardiac origin, where the initial rhythm was ventricular fibrillation or ventricular tachycardia, and the arrest was bystander witnessed (UK Statistics Authority, 2023). This group, therefore, has a better chance of survival.

The importance of post-ROSC care is well documented (Nolan et al., 2021), with the aim of increasing the proportion of patients who achieve ROSC and then proceed to neurologically intact survival. A post-ROSC ‘bundle of care’ was introduced to UK ambulance services in April 2018 by NHS England (NHS England, 2018). This bundle outlines the minimum treatment regime that should be administered once ROSC is achieved. The evidence underpinning this bundle is derived from international guidelines provided by organisations such as the European Resuscitation Council and the American Heart Association, as well as pivotal research studies demonstrating the benefits of comprehensive post-resuscitation care, including targeted temperature management, haemodynamic optimisation and timely coronary reperfusion.

Additionally, national clinical guidelines from the Resuscitation Council UK and clinical audit data from UK ambulance services contribute to the evidence base, ensuring that the practices recommended are robust and effective. Ambulance services throughout the UK supply data directly to NHS England to monitor their performance according to the national ‘Ambulance Quality Indicators’ (AQIs). The AQIs were introduced in April 2011 in England to monitor ambulance response times and the quality of care provided by ambulance services. The AQIs are ambulance-specific and designed to be consistent with measures in other parts of the NHS, such as emergency departments. The AQIs that focus on clinical outcomes include the post-ROSC bundle.

The AQIs provide a standardised method for measuring and comparing the performance of ambulance services across the country. They also help to identify areas for improvement and ensure that patients receive high-quality care and treatment. The AQIs are regularly reviewed and updated to reflect changes in clinical practice and advances in technology.

The South East Coast Ambulance Service NHS Foundation Trust (SECAmb) has developed the critical care paramedic (CCP) role to provide additional advanced pre-hospital care. CCPs are experienced paramedics who have undertaken significant postgraduate study, including in-hospital and work-based placements. They have 24-hour access to advice from a remote consultant physician to support them in patient treatment and management. An enhanced governance system, including case review and frequent protected skills assurance time, permits the delivery of an enhanced scope of practice. In the context of this AQI, none of the required therapies falls into this extended scope of practice, but it is expected that, due to their frequent exposure to high-acuity incidents such as cardiac arrest, CCPs are well placed to ensure that this minimum treatment regime is delivered.

The benefit of specialist paramedics in the care of high-acuity patients seems intuitively positive but remains contentious in current evidence (Cowley et al., 2019; von Vopelius-Feldt et al., 2014, 2020). This retrospective cohort study shows how CCPs within one semi-urban ambulance trust may benefit patients who are successfully resuscitated, increasing their chance of neurologically intact survival. Given the findings that CCPs improve compliance with the post-ROSC bundle, it is plausible that their advanced training, frequent exposure to high-acuity situations and structured support systems could also enhance compliance with other bundles of care. Future research should explore this potential and investigate the subsequent impact on patient outcomes across various care protocols.

SECAmb covers an area of 3600 square miles, with the population varying between dense urban areas and sparse rural areas. The Trust has over 4000 staff across 110 sites. At the time of this study, there were 78 CCPs employed by SECAmb who provide a 24/7 service based at 10 regional hubs. They are dispatched by a dedicated critical care desk staffed by a CCP. In the context of OHCA, dispatch is at the discretion of the critical care desk operative, but CCPs are often targeted to these incidents due to the enhanced post-ROSC package they can deliver, as well as to provide senior clinical support during the resuscitation attempt.

The majority of CCPs within SECAmb currently operate as specialist paramedics, but specialist and advanced paramedic roles vary significantly across UK ambulance trusts, with differing titles, training standards and scopes of practice. This lack of standardisation complicates comparisons across trusts and underscores the need to contextualise findings. Previous studies, such as Coppola et al. (2021), have demonstrated the ways in which diverse care models can influence outcomes in emergency settings.

### Aims and objectives

The aim of the study was to determine whether CCP attendance at OHCA improves the delivery of a post-ROSC bundle of care.

Data from 12 months’ worth of post-ROSC patients (approximately 1000) within the Trust were analysed and categorised according to the presence (or absence) of a CCP at the scene. The primary outcome was full compliance with the prescribed post-ROSC care bundle.

Secondary outcomes were to identify compliance within the separate therapeutic constituents, allowing for identification of areas to target for improvement.

## Methods

### Study design

This study complied with the Strengthening the Reporting of Observational Studies in Epidemiology (STROBE) guidance, which is the recommended reporting tool for observational cohort studies (von Elm et al., 2007).

The data used for this study were routinely collected as part of the patient care record (PCR). This was electronically collated by the trust and then made available to the Trust’s clinical audit team, who supplied the required parameters to NHS England. The study included sequential patients presenting with OHCA and ROSC from 1 September 2021 to 31 August 2022. The categories of data that were gathered for the study are shown in Table 1.

**Table 1.** Categories of data collected.

Category	Notes
Age	
Sex	
ROSC at any time**?**	Yes/No.
Survival to 30 days**?**	Exclude patients for whom survival at 30 days is not known; for example, those unavailable via the Summary Care Record application (SCRa).
12-lead ECG**?**	Part of post-ROSC care bundle (Table 2). Yes/No.
Capillary blood glucose **(**CBG**)?**	Part of post-ROSC care bundle (Table 2). Yes/No.
Blood pressure**?**	Part of post-ROSC care bundle (Figure 2). Yes/No.
End-tidal carbon dioxide (EtCO_2_**)** capnography**?**	Part of post-ROSC care bundle (Table 2). Yes/No.
Oxygen administered**?**	Part of post-ROSC care bundle (Figure 2). Yes/No.
Fluids**?**	Part of post-ROSC care bundle (Table 2). Yes/No.
Post-ROSC care bundle compliant**?**	Yes/No. If, for any component, no exceptions apply, and the component is not delivered, the care bundle is *not* delivered. If each component is either met or has a valid exception, the care bundle is delivered.
CCP on scene**?**	Yes/No.

### Minimum sample size

No previous similar research exists on which to base effect size and standard deviation estimates. Using standard parameters (α = 0.05, β = 0.2), we were able to detect a moderate effect (0.24) with a sample size of 500, based on a power of 0.80. Since this effect is considered clinically relevant, 500 participants is a conservative estimate to detect a statistically significant result.

### Inclusion criteria

Any patient who had resuscitation (advanced or basic life support) commenced or continued by the ambulance service following an OHCA, and had ROSC on scene, between the dates of 1 September 2021 and 31 August 2022 was considered for inclusion. This study specifically examined care provided in a UK trust that employs CCPs, reflecting the advanced scope of practice unique to these roles.

The study excluded:

- traumatic cardiac arrest;
- patients successfully resuscitated before the arrival of ambulance staff;
- ROSC achieved en route to or upon arrival at the hospital;
- patients aged less than 18 years.

The inclusion and exclusion criteria were consistent with those outlined in the AQI requirements (NHS England, 2018).

In terms of the individual care components, each has its own exceptions, and these are detailed in Table 2.

**Table 2.** Post-ROSC care bundle components (NHS England, 2021).

Component of post-ROSC care bundle	Exceptions	Comment
12-lead ECG **(**taken post ROSC**)**	Patient refusal. Patient re-arrested with ROSC <10 minutes in duration.	If the patient is in arrest on arrival, assume that 12-lead ECG is post ROSC.
CBG recorded post ROSC	Patient refusal. Patient re-arrested with ROSC <10 minutes in duration. CBG measured prior to ROSC and found to be within normal range.	If CBG pre-ROSC is below normal range, a subsequent CBG is required.
EtCO_2_ reading / waveform recorded post ROSC / continuously	Patient refusal. Patient re-arrested with ROSC <10 minutes in duration. Not required: no advanced airway in situ.	
Oxygen administered post ROSC / continuously	Patient refusal. Patient re-arrested with ROSC <10 minutes in duration. Not required: oxygen saturations were 94–98% (88–92% in the case of chronic obstructive pulmonary disease).	
Systolic blood pressure reading recorded post ROSC or, if unobtainable, presence of radial pulse documented	Patient refusal. Patient re-arrested with ROSC <10 minutes in duration.	
Administration started of a 250 ml bolus of saline fluids post ROSC	Patient refusal. Patient re-arrested with ROSC <10 minutes in duration. Not required: systolic blood pressure >90 or presence of a radial pulse where blood pressure is unobtainable, evidence of significant heart failure or hypervolaemia clearly documented. All attempts to gain intravenous and intraosseous vascular access are unsuccessful.	A flush of 10 ml is not considered, as fluids administered.

### Data analysis

The primary outcome was assessed using the categories ‘Post-ROSC care bundle compliant’ and ‘CCP on scene’. This was in the form of binary data (Yes/No) and allowed an initial comparison between the ‘CCP’ and ‘non-CCP’ groups in terms of the proportion of incidents that were compliant. Initially, a chi-square test of independence was selected to determine the significance of group differences. However, limitations in the chi-square test’s applicability were identified due to the potential imbalance in group distributions and sample size disparity. This necessitated the use of the Mann-Whitney U test as a non-parametric alternative to assess differences in compliance rates.

The study involved two groups with a notable size discrepancy: 891 incidents in the CCP group and 106 incidents in the non-CCP group, resulting in a nearly 1:9 ratio between the groups. This imbalance may impact the validity of certain statistical models and should be considered when interpreting the results. The analysis was conducted with these considerations in mind, and non-parametric tests, such as the Mann-Whitney U test, were employed to mitigate the effects of the unequal sample sizes.

Before performing the Mann-Whitney U test, assumptions were reviewed to ensure its appropriateness. These include the independence of observations, the ordinal nature of the data and that the distributions of compliance percentages across groups were not significantly different in shape, as assessed visually and through statistical tests (e.g. Kolmogorov-Smirnov). Any potential discrepancies in distribution were noted as limitations.

Calculating the mean percentage total compliance (i.e. what percentage of the bundle was completed from the six elements) allowed for a more detailed analysis of the primary outcome. Normality was assessed numerically using a chi-squared goodness-of-fit model, but due to the nature of the data, it was expected that the data would not fit a normal distribution. This was due to an expected high proportion of ‘Yes’ results, which would all return a 100% completion rate. Due to the likely skewed distribution, an unpaired t-test could not be used, so a Mann-Whitney U test was deemed the most appropriate measure of the central tendencies. Due to the discrete nature of the percentage data (100%, 83.33%, 66.67%, 50%, 33.33%, 16.67% or 0% are the only options), a large number of ties were anticipated. A modified U number was used in order to account for the tied data (McGee, 2018).

In terms of secondary outcome, each of the care bundle components was separately analysed to determine how often each were completed within the CCP and non-CCP groups. These figures were displayed along with relative risk, 95% CI and chi-square test of independence.

Separately, descriptive data (e.g. demographics) were displayed in both graphical and tabular form. All data processing was done using Microsoft Excel software.

## Results

### Participants

**Figure 1:**
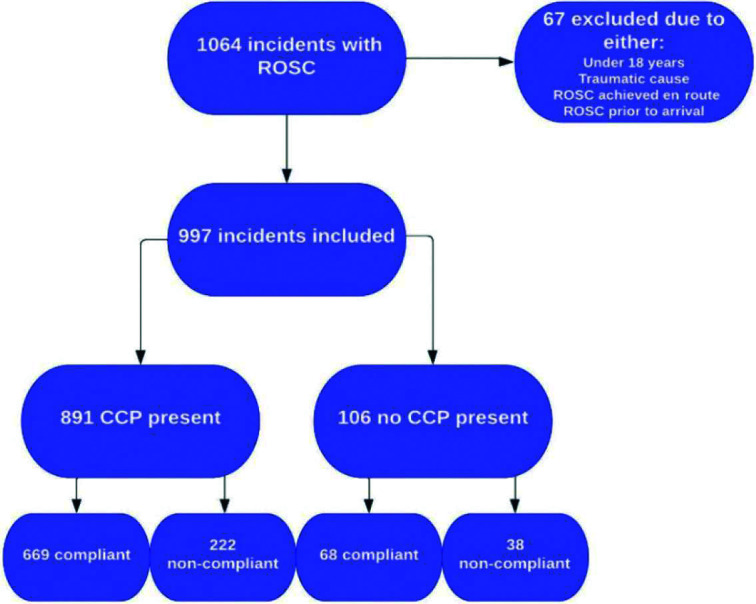
Figure 1. Study flowchart.

In the study period, SECAmb attended 1064 incidents where ROSC was obtained at the scene. Of these, 67 met one of the NHS England exclusion criteria, leaving 997 incidents for inclusion. Patient characteristics and the study flowchart are available to view in Table 3 and Figure 1, respectively. Missing data can be broken down into two types:

**Table 3.** Summary of patient characteristics.

	All	CCP	Non-CCP
Age (mean)	64.52	64.57	69.49
% male vs female	66 v. 33	64 v. 36	71 v. 29
% Utstein	21	27.6	10.6

- Missing patient clinical record – all clinical records were accounted for.
- Missing outcome data – where outcome data was not recorded, it was assumed to have not been done.

Of the 997 included incidents, 644 were male (66%) and 353 female (34%). The mean age was 64.52 years. A CCP attended 891 (89%) of the incidents.

Analysis of the data shows that where a CCP was present, 669 incidents (75%) were marked as fully compliant with the ROSC bundle, compared with 68 (64%) of incidents without a CCP. This is a statistically significant finding and results in a relative risk (in terms of benefit) of 1.17 (95% CI 1.01‒1.36, p = 0.036).

The number needed to treat (NNT) is 9.15 (95% CI 5.06‒47.2), indicating that for every 9.15 cases in which a CCP is present, one additional patient is likely to receive full compliance with the post-ROSC care bundle. The NNT is a useful metric in this context, highlighting the potential impact of CCP involvement on improving adherence to post-resuscitation care. While the NNT provides a sense of how many patients would need to be treated with CCP support to see one additional compliant case, its interpretation should consider the severity of patient outcomes, the potential side effects and the costs associated with deploying CCPs.

**Figure 2:**
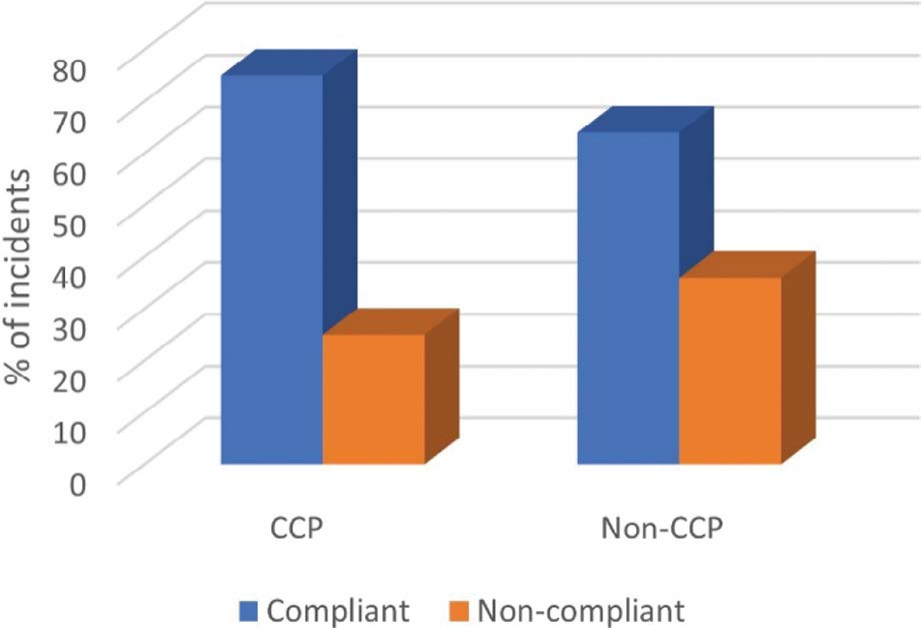
Figure 2. Comparison of compliance outcomes between CCP and non-CCP groups. Relative risk of benefit 1.17 [1.01–1.36] vs 0.85 [0.74–0.99] (p <0.05, chi-square test). NNT = 9.15 [47.2–5.06]. Total percentage compliance 75.69% vs 65.25% (p <0.001), Mann Whitney-U test. Goodness-of-fit test demonstrated non-normality (p <0.05).

When examining the total mean percentage compliance across the six standards, the CCP group showed a statistically significant positive result (75.69% [SD 0.21] vs 65.25% [SD 0.24], p <0.001). The chi-square test was initially applied to determine group differences; however, preliminary results indicated that distribution assumptions were not met (e.g. imbalance in sample sizes and non-normal data). Consequently, a Mann-Whitney U test was performed. Non-normality was confirmed using a Kolmogorov-Smirnov test (p <0.05), and distributions were further reviewed to ensure the appropriateness of the non-parametric approach. Statistical analyses followed best practices for non-parametric testing, including verification of assumptions for the Mann-Whitney U test (McGee, 2018). This is displayed in Figure 2.

**Figure 3:**
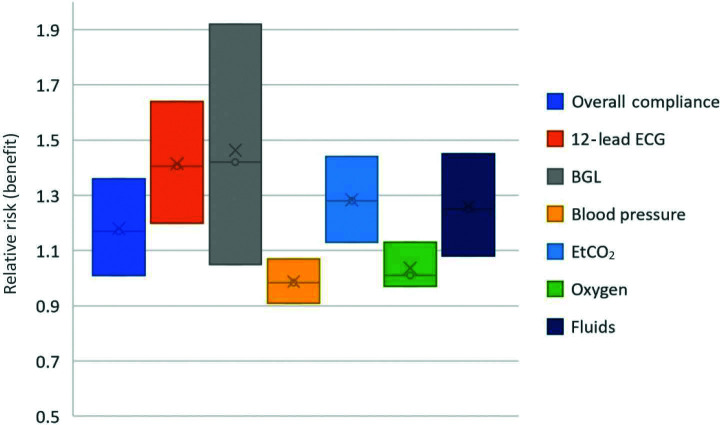
Figure 3. Relative risk (of benefit) for CCP presence.

Secondary outcome analysis focussed on the individual care bundles. These are displayed in Table 4 and Figure 3. EtCO_2_ had the highest compliance rate in the CCP group (91.58%, SD 0.03), whereas blood pressure performed best in the non-CCP group (86.79%, SD 0.34). CBG showed the poorest compliance in both groups (CCP 42.87% [SD 0.02] vs non CCP 30.19% [SD 0.46]). Statistically significant benefits were shown for compliance in the CCP group for 12-lead ECG (RR 1.405, p <0.01), CBG (RR 1.42, p = 0.02), EtCO_2_ (RR 1.28, p = 0.001) and fluids (RR 1.25, p = 0.0027).

**Table 4.** Secondary outcome in percentage compliance across the six standards.

	CCP	Non-CCP	P-value	RR [95% CI]	NNT [95% CI]
12-lead ECG, mean % [SD, 95% CI]	75.31 [0.43, 0.01]	60.38 [0.49, 0.01]	<0.001	1.41 [1.2–1.64]	4.09 [3.18–5.95]
CBG mean **%** [SD**,** 95% CI]	42.87 [0.02, 0.004]	30.19 [0.46, 0.01]	0.02	1.42 [1.05–1.92]	7.884 [4.43–35.81]
Blood pressure, mean **%** [SD, 95% CI]	85.41 [0.03, 0.006]	86.79 [0.34, 0.01]	0.69	0.98 [0.91–1.07]	72.37 [11.82–17.85]
EtCO_2_, mean** % **[SD, 95% CI]	91.58 [0.03, 0.0006]	71.70 [0.45, 0.01]	0.001	1.28 [1.13–1.44]	5.06 [3.87–7.31]
Oxygen, mean **% **[SD, 95% CI]	78.23 [0.03, 0.006]	77.36 [0.42, 0.01]	0.84	1.01 [0.97–1.13]	115.12 [10.9–213.41]
Fluids, mean % [SD, 95% CI]	80.25 [0.03, 0.0006]	64.15 [0.48, 0.01]	0.003	1.25 [1.081–1.45]	6.213 [4.115–12.67]

The analysis reveals a statistically significant difference in post-ROSC care compliance between the CCP and non-CCP groups. However, it is important to note the substantial size discrepancy between the two groups (nearly 9:1), which may influence the robustness of the results. The use of non-parametric tests was chosen to address this issue and ensure the validity of the findings despite the unequal group sizes.

### Discussion and limitations

This study demonstrates that the presence of a CCP at an incident where ROSC was obtained at the scene following OHCA was associated with a statistically significant increase in compliance with the ROSC bundle. Of the incidents with a CCP present, 75% were fully compliant with the ROSC bundle, compared with 64% of incidents without a CCP. The study also found that the mean percentage compliance across the six standards was significantly higher in the CCP group than in the non-CCP group. This suggests that the presence of a CCP may have a positive impact on overall compliance with the ROSC bundle. The secondary outcome analysis focusing on individual care bundles further supports this, showing statistically significant benefits in compliance for several care bundles when a CCP was present. Demographic data shows a prevalence of males, above that of the national demographic, but this is consistent with many studies that have identified an apparent sex bias within cardiology (Regitz-Zagroesk et al., 2016), those examining ROSC patients exclusively (Awad et al., 2021) and prior studies within the Trust (Cowley et al., 2021).

The study identified differences in compliance rates between individual care bundles, with EtCO_2_ having the highest compliance rate in the CCP group. This aligns with its critical role in assessing ventilation and perfusion adequacy during post-ROSC care, as emphasised by the Resuscitation Council UK’s ABCDE approach. Blood pressure assessment was performed most frequently in the non-CCP group, while CBG, a less critical metric in this context, showed the poorest compliance across both groups. These findings underscore the need to prioritise interventions such as EtCO_2_ monitoring, which directly inform clinical decision-making and patient outcomes.

The study was conducted in a single ambulance service in the UK with a specific model of specialist practice, and therefore the findings may not be generalisable to other settings. The absence of uniform national standards for advanced or senior paramedic roles presents challenges in generalising these findings beyond this trust. While this study highlights the impact of CCPs within one trust, further research should explore how variations in role definitions, training and resource allocation across UK trusts influence compliance with post-ROSC bundles and other care protocols. Methodologies such as those used by Coppola et al. (2021) could be valuable in addressing this complexity. Other areas of limitation, particularly due to information and confounding bias, have already been discussed.

The enhanced compliance observed with the ROSC bundle suggests that CCPs might also achieve better compliance with other care bundles, given their advanced training and frequent exposure to high-acuity situations. Nevertheless, further research is necessary to confirm these findings, investigate the relationship between compliance with the ROSC bundle and patient outcomes and explore the potential benefits of CCP involvement across various clinical care protocols. Additionally, it is crucial to determine whether the improved compliance is due to the specialist skills of CCPs or simply to the presence of an additional person on the scene. Addressing these questions will help to better understand the true impact of CCPs on patient care and outcomes.

While this study demonstrates that CCPs significantly improve compliance with the post-ROSC care bundle, it is essential to consider the broader role of CCPs in patient outcomes. If the CCP role primarily serves an administrative compliance function rather than directly improving outcomes, future research should focus on optimising CCP interventions to enhance survival and neurological recovery. In particular, the study’s focus on compliance with the 4 Hs and 4 Ts (hypoxia, hypovolaemia, hypothermia, hypoglycaemia, toxins, thrombosis, tension pneumothorax and tamponade) emphasises the importance of foundational care, but additional interventions, such as vasopressors like adrenaline, should also be explored.

While fluid administration was a key compliance indicator, the role of vasopressors in post-ROSC care, especially for patients with high-risk profiles (such as those identified by the Utstein criteria), should be considered in future studies. The next steps in this field should move beyond administrative compliance to investigate the impact of these advanced interventions on patient outcomes. Understanding how CCP involvement, alongside other treatments, influences survival and neurological recovery will be essential for refining post-ROSC care strategies.

A limitation of this study is the significant size discrepancy between the two groups, with the CCP group comprising nearly nine times as many incidents as the non-CCP group. This imbalance could limit the validity of some statistical models and may affect the interpretation of results. Future studies with more balanced group sizes would help validate these findings and enhance the generalisability of the conclusions.

This study was conducted within a single ambulance trust that employs CCPs. The findings may not be directly transferable to other trusts where advanced paramedic roles and care models differ significantly.

The data used in this study was routinely collected as part of the patient’s care record, which could be subject to errors or omissions. There could be missing or incomplete data, which may impact the accuracy of the results. However, there is no pre-study evidence to suggest CCPs are any better or worse at completing documentation, so this bias would not be inherently greater in either of the two groups and so is not anticipated to bias any comparator conclusions, only the overall numbers. In addition, the data was collected by the Trust’s clinical audit team, which may introduce bias if they are not fully independent and objective.

There is no anticipated bias in the primary outcome measure from the discretionary dispatch of a CCP to cardiac arrest. While this may mean that CCPs were ‘targeted’ to more viable resuscitation attempts, this would affect the likelihood of obtaining a ROSC, but would not affect the delivery of a care bundle once ROSC was achieved. It should be considered a significant source of bias if examining whether CCPs *improve* the *likelihood* of ROSC and associated outcome data (such as 30-day survival).

Another critical question that arises is whether the improved compliance observed with the ROSC bundle is due to the specialist paramedics (CCPs) genuinely influencing the treatment or simply being more diligent in recording whether interventions were performed or providing reasons for not doing so. Furthermore, if CCPs did influence the treatment, it is essential to determine whether this was attributable to their specialist training and skills or merely the result of having an additional person on the scene. These factors introduce potential biases and confounding variables that should be addressed in future research to better understand the true impact of CCPs on patient outcomes.

However, the results presented in the study are promising and suggest that the presence of a CCP during incidents of ROSC can improve compliance with the ROSC bundle, potentially leading to better outcomes for patients. Further research with larger sample sizes and in different settings is needed to confirm these findings.

It is important to note that the study did not examine patient outcomes; it is thus unclear whether the improved compliance translated to better patient outcomes. Future research should investigate the relationship between compliance with the ROSC bundle and patient outcomes.

Another potential limitation is the risk of Type II error being introduced by distribution differences between groups, which could impact the sensitivity of the Mann-Whitney U test. Efforts were made to assess and account for these differences through visual inspection and statistical testing prior to analysis. Nevertheless, the possibility of residual bias remains and warrants consideration in future studies.

In summary, the results of this study indicate that the presence of a CCP during incidents of ROSC can significantly improve compliance with the ROSC bundle, particularly for specific care elements. These findings have important implications for the delivery of pre-hospital care and underscore the potential advantages of integrating CCPs into the pre-hospital care team. The post-ROSC bundle introduced by NHS England is based on international guidelines, key research studies and national clinical guidelines, which collectively emphasise the importance of comprehensive post-resuscitation care. However, it is important to acknowledge that there is no direct evidence that the post-ROSC bundle itself improves patient outcomes.

## Conclusion

In conclusion, this study provides evidence that the deployment of a CCP to incidents where ROSC has been achieved can increase compliance with the ROSC bundle and individual care components. While the findings suggest that CCPs may have a positive impact on compliance, it is important to note that this study does not demonstrate direct improvements in patient outcomes. Identifying areas of lower compliance can help target interventions to improve care, but further research is needed to assess whether CCP involvement tangibly enhances patient survival and neurological recovery.

Additionally, while compliance with metrics such as EtCO_2_ is crucial for guiding resuscitation efforts, metrics such as CBG should be viewed in context, as their role is secondary in post-ROSC care. Future research should explore the potential benefits of advanced interventions, including vasopressors such as adrenaline, and examine how CCPs can contribute to improving patient outcomes, particularly in high-risk cases as defined by the Utstein criteria.

Ultimately, this study underscores the potential benefits of CCPs in improving compliance with the post-ROSC care bundle but highlights the need for further investigation into their impact on patient outcomes and the role of additional interventions.

## Acknowledgements

The authors acknowledge the contribution made by Eleanor Jaquet (Cardiac Arrest Analyst) and Sophie Clark (Quality Improvement Lead) of South East Coast Ambulance Service NHS Foundation Trust for their assistance in support of the project and data collation.

## Author contributions

AC was responsible for conceptualisation, methodology, resourced, data curation, formal analysis, investigation, writing of the original draft and review and editing of the article. DC undertook formal analysis and review and editing of the article. PR was responsible for conceptualisation, writing of the original draft, review and editing of the article and supervision. AC acts as the guarantor for this article.

## Conflict of interest

None declared.

## Ethics

This study met UK Health Research Agency criteria for a service evaluation. All the data utilised for this study were routinely collected as part of standard pre-hospital patient data collection. A full DPIA was performed and approved by the Trust’s IG department. Formal ethical approval was therefore not required. This study meets the requirements of the Strengthening Reporting of Observational Studies in Epidemiology (STROBE) checklist.

## Funding

None.

## References

[bibr_1] Awad E. Humphries K. Grunau B. et al (2021). The effect of sex and age on return of spontaneous circulation and survival to hospital discharge in patients with out of hospital cardiac arrest: A retrospective analysis of a Canadian population. *Resuscitation Plus*, 5, 100084. https://doi.org/10.1016/j.resplu.2021.100084. 34223350 10.1016/j.resplu.2021.100084 PMC8244242

[bibr_2] Coppola A. Black S. & Endacott R. (2021). How senior paramedics decide to cease resuscitation in pulseless electrical activity out of hospital cardiac arrest: A mixed methods study. *Scandinavian Journal of Trauma, Resuscitation and Emergency Medicine*, 29(1), 138. https://doi.org/10.1186/s13049-021-00946-7. 34530872 10.1186/s13049-021-00946-7 PMC8447587

[bibr_3] Cowley A. Cody D. & Nelson M. (2021). The epidemiology and effectiveness of synchronized cardioversion in a UK prehospital setting: A retrospective cross-sectional study. *Prehospital and Disaster Medicine*, 36(4), 440–444. https://doi.org/10.1017/S1049023X21000546. 34127157 10.1017/S1049023X21000546

[bibr_4] Cowley A. Durham M. Aldred D. et al (2019). Presence of a pre-hospital enhanced care team reduces on scene time and improves triage compliance for stab trauma. *Scandinavian Journal of Trauma, Resuscitation and Emergency Medicine*, 27(1), 86. https://doi.org/10.1186/s13049-019-0661-z. 31492193 10.1186/s13049-019-0661-z PMC6731599

[bibr_5] McGee M. (2018). Case for omitting tied observations in the two-sample t-test and the Wilcoxon-Mann-Whitney Test. *PLoS One*, 13(7). https://doi.org/10.1371/journal.pone.0200837. 10.1371/journal.pone.0200837 PMC6057651 30040850

[bibr_6] NHS England. (2018). *Ambulance quality indicators*. NHS England. https://www.england.nhs.uk/statistics/statistical-work-areas/ambulance-quality-indicators/.

[bibr_7] NHS England. (2021). *Ambulance quality indicators: Clinical outcomes specification – Post-ROSC care bundle components*. Version 1.0. https://www.england.nhs.uk/statistics/wp-content/uploads/sites/2/2021/06/20210610-AmbCO-specification.pdf.

[bibr_8] Nolan J. P. Sabdroni C. Bottiger B. W. et al (2021). European Resuscitation Council and European Society of Intensive Care Medicine Guidelines 2021: Post-resuscitation care. *Resuscitation*, 161, 220–269. https://doi.org/10.1016/j.resuscitation.2021.02.012. 33773827 10.1016/j.resuscitation.2021.02.012

[bibr_9] Regitz-Zagroesk V. Oertelt-Prigione S. Prescott E. et al (2016). Gender in cardiovascular diseases: Impact on clinical manifestations, management, and outcomes. *European Heart Journal*, 37(1), 24–34. https://doi.org/10.1093/eurheartj/ehv598. 26530104 10.1093/eurheartj/ehv598

[bibr_10] UK Statistics Authority. (2023). *Statistical note: Ambulance quality indicators (AQI)*. NHS England. https://www.england.nhs.uk/statistics/wp-content/uploads/sites/2/2023/05/20230511-Stats-Note-AQI-1.pdf.

[bibr_11] von Elm E. Altman D. G. Egger M. et al (2007). The Strengthening the Reporting of Observational Studies in Epidemiology (STROBE) statement: Guidelines for reporting observational studies. *The Lancet*, 370(9596), 1453–1457. https://doi.org/10.1016/S0140-6736(07)61602-X. 10.1016/S0140-6736(07)61602-X 18064739

[bibr_12] von Vopelius-Feldt J. Morris W. & Benger J. (2020). The effect of prehospital critical care on survival following out-of-hospital cardiac arrest: A prospective observational study. *Resuscitation*, 146, 178–187. https://doi.org/10.1016/j.resuscitation.2019.08.008. 31412291 10.1016/j.resuscitation.2019.08.008

[bibr_13] von Vopelius-Feldt J. Wood J. & Benger J. (2014). Critical care paramedics: Where is the evidence? A systematic review. *Emergency Medicine Journal*, 31(12), 1016–1024. https://doi.org/10.1136/emermed-2013-202721. 24071949 10.1136/emermed-2013-202721

